# Plaque Psoriasis Exacerbation and COVID-19 Vaccination: Assessing the Characteristics of the Flare and the Exposome Parameters

**DOI:** 10.3390/vaccines12020178

**Published:** 2024-02-09

**Authors:** Emmanouil Karampinis, Myrto-Maria Papadopoulou, Kleoniki Chaidaki, Konstantina-Eirini Georgopoulou, Stavroula Magaliou, Angeliki Viktoria Roussaki Schulze, Dimitrios P. Bogdanos, Efterpi Zafiriou

**Affiliations:** 1 Department of Dermatology, Faculty of Medicine, School of Health Sciences, University General Hospital of Larissa, University of Thessaly, 41110 Larissa, Greece; ekarampinis@uth.gr (E.K.); kleonikichaidaki@gmail.com (K.C.); roussaki@otenet.gr (A.V.R.S.); 2 Department of Internal Medicine, General Hospital of Karditsa, 43131 Karditsa, Greece; myrto.papadopoulou4@gmail.com; 3 Department of Dermatology, General Hospital of Nikaia Pireus “Agios Panteleimon”—General Hospital of West Attica “Agia Varvara”, 12351 Athens, Greece; 4 Department of Internal Medicine, General Hospital of Trikala, 42100 Trikala, Greece; 5 Department of Rheumatology and Clinical Immunology, Faculty of Medicine, School of Health Sciences, University General Hospital of Larissa, University of Thessaly, 41110 Larissa, Greece; bogdanos@med.uth.gr

**Keywords:** COVID-19, vaccination, plaque psoriasis, exposome, flare

## Abstract

The diverse patient population and widespread vaccination in the COVD-19 era make vaccine-triggered episodes of psoriasis an ideal model of exposome research. This scenario explores the fine balance between protective and exacerbating factors, providing insights into the complex relationship between environmental exposure and psoriasis immunopathogenesis when a trigger appears, such as that of the hyperinflammatory state induced by the COVID-19 vaccine. Analyzing interactions between vaccine-induced phenomena and exposome parameters may provide clinically relevant information important for personalized medicine decision-making. We performed a literature review seeking patients with plaque psoriasis flares or new onset or change in plaque psoriasis into another psoriasis subtype, such as pustular or erythrodermic flare, focusing on the inner and external exposome traits of patients. We identified 71 patients with plaque psoriasis flares, 12 patients with new-onset psoriasis, and 17 with plaque psoriasis subtype change, and assessed the COVID-19 vaccine-induced plaque psoriasis in terms of clinical presentation, post-vaccination flare period and treatment status, as well as inner exposome parameters (genomics, oxidative stress, hormonal impact due to gender, aging, skin color) and external parameters (UV, infectomics). Novel data on psoriasis flares following COVID-19 vaccination are primarily obtained by combining exposome and vaccine-triggered episode features and characteristics and comparing them with similar psoriasis flares unrelated to COVID-19 vaccination.

## 1. Introduction

During the early stages of public vaccinations, the occurrence of cutaneous side effects associated with COVID-19 vaccination was noted, encompassing various dermatological conditions, such as the exacerbation of inflammatory dermatosis as psoriasis and the reactivation of skin infections including herpes zoster [1]. Over time, a multitude of case reports, case series, and case–control studies emerged worldwide, uncovering instances of new-onset diseases or exacerbations that developed following vaccination, while reviews on the same topic attempted to offer mechanistical explanations and to identify potential connection links between the immune response after COVID-19 vaccination and the skin manifestations presented, without being able to clarify the specific triggering elements implicated in skin flares [2]. Most studies raised hypothetical scenarios rather than coherent data and sound explanations of the observations recorded.

COVID-19 vaccination stood as one of the most secure and economically efficient public health measures for preventing and managing COVID-19. There are predominantly five categories of vaccine subtypes developed and utilized for vaccination, comprising whole virus types (live attenuated and inactivated), viral vectors (replicating and non-replicating, such as Johnson & Johnson’s, Janssen, and AstraZeneca), protein subunit vaccines, nucleic acid options (DNA and RNA), and virus-like particle (VLP) vaccines. The most popular COVID-19 vaccines included messenger RNA (mRNA) (Pfizer-BioNTech and Moderna) [3]. The majority of COVID-19 vaccines have been formulated to induce immune responses, particularly in antibodies that target the spike protein of the SARS-CoV-2 virus, blocking the virus’ entry into the host cells (neutralization) [4]. Apart from this function, antibody-dependent cellular phagocytosis depends on opsonization, and antibody-dependent cellular cytotoxicity and complement deposition are part of the coordination between antibodies and cellular responses, particularly those involving CD4+ and CD8+ T cells caused by the vaccine [5]. Nevertheless, different types of vaccines have not demonstrated uniformity in generating immune responses. Adenoviral COVID-19 vaccines, like AstraZeneca, ChAdOx1, and J&J Ad26.COV2.S, can provoke Th1and CD8 T cell responses, while inactivated virus COVID-19 vaccines, such as CoronaVac and Covaxin (BBV152), induce comparatively modest CD4 T cell responses and a combination of Th1 and Th2 cells as well as a weak CD8 response. However, Covaxin might be more efficacious due to significant Th1 responses [3]. Studies also indicate a decline in immunity from COVID-19 vaccines over time, with this reduction being more noticeable in older individuals and those with certain underlying health conditions, such as immunodeficiencies. Considering the fact that a booster dose of the COVID-19 vaccine can elicit a strong antibody response that could protect the individuals from acquiring severe disease, it is evident that booster doses continue to play an important role [6]. Apart from the beneficial role, host responses to immune responses triggered by COVID-19 vaccination included systemic immune activation at times accelerated to an extent resembling a cytokine storm, with the production of various pro-inflammatory cytokines that participate in the immunopathogenesis of many inflammatory skin disorders, such as psoriasis vulgaris [7].

Patients with psoriasis are considered a priority for COVID-19 vaccination due to the frequently accompanying comorbidities and their treatment-induced immunosuppressive status, while in case of infections, a more severe clinical outcome is often anticipated. Also, another reason why vaccination is important is that psoriasis patients on immunosuppressive medications are at increased risks of post-infectious complication potentially including more severe forms of SARS-CoV-2 infection. Treatment modalities, like interleukin (IL)-17 inhibitors, fundamental modulatory agents for the efficacious treat psoriasis, have the potential to compromise mucosal immunity, consequently elevating the susceptibility to upper and lower respiratory tract infections [8]. Additionally, anti-tumor necrosis factor (anti-TNF) agents and other immunosuppressive medications, like methotrexate and cyclosporine, occasionally employed in psoriasis management, may also heighten the risk of pulmonary infections, including SARS-CoV-2 [9].

Patients with chronic inflammatory dermatosis, such as psoriasis, usually ask about the safety of COVID-19 vaccination and if it will affect the course of their disease, as psoriasis flares have been reported. Indeed, psoriasis flares following vaccinations, also called “psoriasis vaccinalis”, are not a new observation, as it has been reported after other vaccines as well including BCG and influenza [10]. However, in the case of non-COVID-19 vaccines, guttate psoriasis was the most frequently psoriasis subtype encountered. Flares and new-onset exacerbations of plaque psoriasis were noted by studies published in the medical literature, as well as a subtype modification, changing from plaque psoriasis to another form, such as pustular psoriasis or erythrodermic psoriasis. In this case, due to differences in the pathogenesis of the different psoriasis forms, it was implied that the immune response mounted by the vaccine could modify the main features of the disease, including the reservoir of the predominant cytokine and the clinical presentation of the skin characteristics. For example, in the case of plaque psoriasis, IL-17 and IL-22 contribute to the proliferation of skin cells, triggering abnormal skin cell turnover and leading to the thickening and scaling of the skin seen in psoriatic lesions [11], while in pustular psoriasis, the release of excessive amounts of IL-1 and IL-36 results in an inflammatory response in the skin, which leads to the development of pustules [12]. Also, differences between new-onset and chronic plaque psoriasis have been observed, as in the initiation stage, the IL-22/IL-17 axis and activated DCs are the main contributors to the disease, while in chronic disease, mature dermal DCs and T cells contribute more to the cytokine milieu [13]. Due to the immunological differences implicated in new-onset psoriasis, psoriasis flare, and psoriasis subtype modification when a trigger occurs, such as vaccination, it is reasonable to assess the characteristics of the skin flare after the inflammatory contributor, such as the time of occurrence, contributor subtype, and aligned symptoms, as well as the characteristics of the patient, such as the immunology profile and non-vaccine-related exposome traits, such as gender, age, and comorbidities.

The most recent review about psoriasis flares and COVID-19 vaccination was conducted by Potestio et al., who found 49 studies involving 134 patients with psoriasis manifestations, while almost 20% related to new-onset psoriasis [14]. Most of the studies on the topic were case reports and case series reporting on the post-vaccination period of the flare, the type and dose of the vaccine, and the treatment status of the patients—for example, if the patient was undergoing biological treatment or not. Important information, such as comorbidities of the patient or history of COVID-19 infection, were often missing or under-reported. The main conclusive remarks by narrative reviews and larger studies are reported in Table 1.

The diversity of the patients that presented with flare, as well as the universal utilization of vaccines, have the potential to make the vaccine trigger probably one of the best examples to assess exposome factors as well as the fine balance between protective and aggravating factors and to examine how this dynamic scale could be reversed, producing a flare in cases of a triggering factor, such as vaccination.

The term exposome encompasses all environmental elements that an individual encounters throughout their lifetime, referring to the cumulative exposure to various environmental factors that can impact human health and ultimately lead to a disease or affect its progression. The exposome is a concept relevant to all skin diseases, including psoriasis. The skin encounters a diverse array of exposures, ranging from lifestyle habits, such as diet, smoking, obesity, sunlight exposure, pre-existing diseases, and exposure to infectious agents, to individual features, like skin microbes, oxidative stress parameters, skin chemical environment, and cutaneous immune reactions. These exposures, in turn, play distinct roles in the pathways implicated in the development of psoriatic skin lesions, shaping the disease course and progression [21].

Genetic factors can be activated by environmental triggers (epigenomics). Those genetic factors can be critical genes involved in systemic and skin immunity, such as SNPs of genes whose products are involved in immune pathways, such as the IL-17/IL-23 axis, as well as skin barrier formation genetic polymorphisms [22]. Systemic oxidative stress conditions correlated perhaps with ageing and the co-existence of other diseases are also another important contributor implicated in many skin disorders [23]. Gender distribution due to the hormonal impact on skin can further affect psoriasis course, as sex hormones, like estrogens, inhibit the production of psoriasis-related cytokines, like IL-1β and IL-23, by neutrophils and dendritic cells, respectively [24]. Metabolics and microbiomes are also inner exposome factors of psoriasis that are being examined in many ongoing studies. A disturbed metabolic profile seems to include patients with obesity and metabolic syndrome, as well as PCOS (polycystic ovary syndrome), diabetes, liver, and thyroid diseases [25]. In skin diseases, variations in skin type and phototype, attributed to diverse pigmentation tones and race-specific skin characteristics, can play a significant role. Discrepancies have been noted in skin cancers and inflammatory skin diseases based on these factors [26].

External exposome factors can also induce a psoriasis flare indirectly by modifying inner exposome factors (for example, oxidative stress triggered by tobacco [27]) as well as direct contributions to the pathogenesis pathways of psoriasis (nicotine stimulates the increased secretion of various cytokines) [28]. External exposome factors include environmental pollution, alcohol and tobacco abuse, as well as stress and sleep deprivation, diet, and lifestyle habits. Epigenetic mechanisms influenced by exposome factors, including DNA methylation, histone modifications, and non-coding RNAs, drive alterations in gene expression, creating susceptibility to psoriasis [21]. Amongst them, some contributors can have beneficial effects on suppressing inflammation, such as a Mediterranean diet and sun exposure. In the case of UV exposure, cytokine profile is linked to psoriasis by steering the immune response away from the proinflammatory production axis. Also, UV radiation, especially UVB, leads to vitamin D production in the skin, which in turn enhances the synthesis of anti-inflammatory cytokines by suppressing or inhibiting the production of proinflammatory cytokines [29]. Another important aspect of exposome is infectomics and autoinfectomics, which are terms used to describe how pathogens may participate in the induction of autoimmunity and the new onset of autoimmune disease, such as psoriasis [30,31]. This omics-section has been studied in COVID-19 infection, too [32].

**Figure 1:**
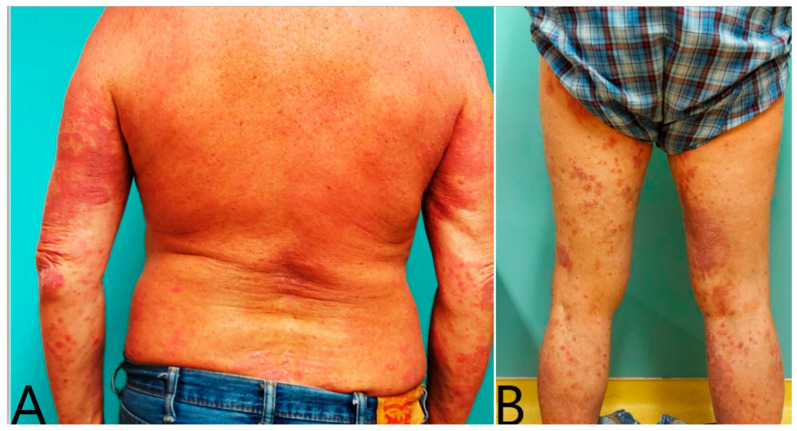
(**A**,**B**) showing a plaque psoriasis exacerbation of a 56-year-old patient 5 days after the 2nd dose of BNT162b2(Pfizer) vaccination. Every patient is unique with factors that predispose towards a flare. The patient has comorbidities, such as hypercholesterolemia and diabetes, and, therefore, a disturbed metabolic profile, as well as a recent stressful event and a medical history of COVID-19 infection in the past 4 months (infectomics).

The sum of Inner and outer exposome contributions create a scale balance to every psoriasis patient, making him/her vulnerable to a psoriasis flare when a trigger is added, by modifying the established inner and external factors pushing towards or away from a flare (Figure 1). For example, an oxidative stress test abnormality was noted in cases of adverse effects caused by COVID-19 vaccination, while the fear and hesitancy towards vaccinations can cause further stress to individuals. Stress due to the hypothalamic–pituitary–adrenal axis leads to an increase in CRH, which contributes to increased levels of neurohormones in the periphery activating immune system cells, such as T lymphocytes, B lymphocytes, and monocytes in peripheral organs, like the skin, leading to cutaneous inflammation, potentially causing a flare-up of psoriasis [33].

Exposome factors can be characterized as two wheels (inner and external exposome factors) that interact with each other, and the dynamic interplay of these two wheels yields the outcome, which is the epigenomic wheel (activation and gene expression whose products suppress or aggregate inflammation parameters of psoriasis), producing more or less systemic and cutaneous inflammation, constituting the primary pathophysiology of psoriasis. Therefore, it is reasonable to study how the addition of a new triggering factor can affect this interplay.

Understanding the balance between psoriasis inner and outer exposome contributions would lead a step further to the development of personalized medicine in psoriasis patients. When choosing to assess exposome scale reverse in cases of COVID-19 vaccination, it is expected that factors, such as oxidative stress or the microbiome, cannot not be studied due to a lack of data from the reports. Also, it is worth mentioning that missing data about other exposome contributors are also to be expected. Finally due to immunopathophysiological differences between new-onset psoriasis, psoriasis flare, and psoriasis subtype modifications, exposome factors in each case would further enlighten the exact cascade of events that the COVID-19 vaccination initiates.

Herein, our thorough review aims to evaluate characteristics of the flare (post--vaccination period, COVID-19 vaccine type, etc.) as well as exposome factors, reported in plaque psoriasis flares, new-onset psoriasis, and subtype psoriasis modifications triggered by vaccination. Investigating the exposome supports the concept of personalized or individualized medicine and the understanding of those variations’ contributions would allow us to tailor healthcare interventions based on specific risk factors for each individual.

## 2. Materials and Methods

We performed a search in the PubMed database including articles published until 1 September 2023. The search was based on relevant terms, such as “Psoriasis flare” OR “Psoriasis new onset” AND “COVID-19 vaccine” OR “SARS-CoV-2 vaccine”. The inclusion criteria were English-based articles that contained adequate information about a plaque psoriasis flare or new onset or change in plaque psoriasis into another psoriasis subtype, such as pustular or erythrodermic flare (GEP), as well as the characteristics of patients (age, sex, ethnicity, etc.). Also, studies that focused on specific exposome characteristics and which had observations on those traits were also included, resulting in a list summarizing the already reported exposome findings. In each case report or study that we reviewed, our focus was on extracting information regarding the time interval between vaccination and symptom flare-ups. We also examined the vaccine dosage, prioritizing the first dose as the primary contributor in cases where mild symptoms appeared after the initial dose and flared after the second dose. Additionally, we analyzed the vaccination subtype, considering factors, such as mRNA, viral vector, or inactivated virus. Furthermore, we sought details on any supplementary symptoms that patients might have developed, including pruritus and arthralgias. A similar search focusing on psoriasis flares after non-COVID-19 vaccinations using terms as “Psoriasis flare” OR “Psoriasis new onset” AND “vaccination” was also performed.

The exposome factors included were as follows:

(1) Sex;
(2) Age;
(3) Comorbidities and, more specifically, the number of comorbidities and chronic diseases, such as hypertension, diabetes, and obesity;
(4) Skin composition differences due to ethnicity and skin color. (In terms of ethnicity, we assessed the origin of the country of the study and we used the term “skin of color” to define non-Caucasian individuals with melanin-rich skin that might belong to several races, including those of Hispanic/Latino, Asian, African, Native American, or Pacific Island origin, as well as mixtures of the different races [26,34]. Also, in cases of a multitude of race populations, such as USA, the race ethnicity was not referred to unless there was an image of the patients flare in the report to assess the skin color of the patient);
(5) Disturbed metabolism. We considered patients with disturbed metabolism to be those who had PCOS, diabetes, obesity, hypercholesterolemia, and thyroid and liver diseases;
(6) UV assessment. As it is challenging to measure the prevalence and intensity of UVR exposure on a population scale, this exposome factor was evaluated using the population attributable fraction (PAF index) of new melanoma cases. Concerning this index, PAFs in each region were calculated as the proportional difference between the estimated number of new cases in 2012—by country/territory, 5-year age group (ages ≥ 30 years), and sex—and the expected number of cases, using incidence rates from a reference population, which is a minimally exposed population, as the melanoma incidence rates are low in dark-skinned African populations with low UVR susceptibility [35]. Differences in new cases are ascribed to the varying levels of UVR exposure between the reference and study populations, and the PAF index is an indicator of the number of cutaneous melanomas attributable to UVR worldwide [36]. For example, concerning melanoma cases in 2012, New Zealand holds the highest PAF index at 0.963, closely followed by Australia with a PAF of 0.961 [35];
(7) Tobacco use. We looked for reported patients as heavy smokers;
(8) Infectomics. We assessed mainly past COVID-19 infection.

Oxidative stress [37,38] and microbiome parameters [39], although very important, would not be assessed, as it is very difficult and not practical to predict those parameters by assessing patients age, gender, comorbidities, and lifestyle choices.

Statistical analysis: Numerical and categorical data were gathered from the reported studies. Comparison of numerical variables between the study groups was performed using Student’s *t*-test for comparing independent samples in two groups when normally distributed and using the Mann–Whitney U-test for independent samples when not normally distributed. Comparison of numerical variables between more than two groups was performed using a one-way analysis of variance test with post hoc multiple two-group comparisons in normal data, and a Kruskal–Wallis test was used for non-normal data. For comparing categorical data, the χ^2^-test was performed. A Shapiro–Wilk test was used to test normality.

## 3. Results

Regarding our assessment, we identified a total of 71 patients that had a medical history of plaque psoriasis and experienced a flare after COVID-19 vaccinations. Apart from the case reports, most of the above-mentioned patients derive from case series, such as Sotiriou et al. [40] and Megna et al. [41], that were the first studies that reported psoriasis exacerbation after COVID-19 immunization. The first study included 14 patients from Greece, while the second study included 11 patients from Italy. We also found 12 cases of plaque new-onset psoriasis and 17 cases of patients with plaque psoriasis modification to another form, such as guttate and pustular psoriasis, making the new-onset category the rarest in the post-COVID-19 vaccination triggered forms.

In the psoriasis subtype modification section, 9 pustular eruptions, 3 generalized erythrodermic, and 5 guttate psoriasis flares occurred in the context of a pro-existing plaque psoriasis. The above-mentioned finding suggests that pustular psoriasis is the most frequently reported psoriasis subtype modification after COVID-19 vaccination (Table 2, Table 3 and Table 4).

The clinical manifestations adhered to the traditional symptoms and characteristic appearance of the diseases in question. The most frequent presentation of the respective flares was generalized erythemato-squamous plaques, while the clinical presentation can be altered with the presences of pustules in the case of pustular psoriasis and systemic symptoms combined with scaling, desquamation, and pruritus in the case of generalized erythrodermic psoriasis. In psoriasis flare-ups, four appeared with psoriatic arthritis, four with nail involvement, and five with pruritus. In the new-onset category, 3 out of 12 experienced pruritus, and none of them experienced psoriasis arthritis or nail involvement, while 2 experienced psoriasis arthritis in the guttate flare. Also, it is worth mentioning that due to limitations in the presentation of cases reports which must adhere to strict instructions by the journals (i.e., a strict word limit), many clinical manifestations could be missed, not giving a completed clinical presentation.

Concerning treatment status, 20 out of 71 patients with flare were under biological treatment, and 4 out of 17 were treated with biological treatment in the psoriasis form change category (2 in guttate forms and 2 in pustular psoriasis forms). As a result, treatment with biological therapy is not significantly associated with a flare or a psoriasis form alterations (*p* = 0.69). Nevertheless, the finding that 28.16% of patients experiencing flares were undergoing biological treatment aligns with previous studies indicating that while biological treatment does not provide absolute protection, it does reduce the likelihood of exacerbations [15,41].

Dosage of vaccine also differed amongst patients with psoriasis flare-ups, with the third dose making its entrance to the last case reports and studies published. The second dose of vaccine was observed in 45 out of 71 patients with flares (63.3%), while 20 out of 71 patients (28.1%) experienced flares after the first dose and only 6 experienced them after the third dose. In the new-onset psoriasis, six patients (50%) presented it after the first dose, five after the second, and one after the third dose. Concerning the subtype change, 4 patients experienced the flare after first dose, 10 after the second dose, and 1 patient after the third dose. Two cases did not report the exact number of doses. Generalized erythrodermic psoriasis occurred only after the second dose, while four pustular psoriasis cases appeared after the first dose and three appeared after the second dose. Performing chi-square tests in those categories did not provide significant associations, probably due to the small cohort sizes.

The vaccine subtypes detected in the flare category were 55 mRNA, 14 viral vector, and 2 inactivated viruses. In the mRNA category, Pfizer and Moderna were the most used vaccines. Eight cases of new-onset psoriasis were attributed to mRNA vaccination, two cases followed the viral vector, and two occurred after the inactivated virus vaccine. Pustular psoriasis flares were attributed in seven out of nine cases (77.7%) with mRNA vaccination. mRNA vaccination was the predominant type in both guttate and general erythrodermic psoriasis. Combining dosing and vaccine, 38 (54%) of the psoriasis flares occurred after the second dose of an mRNA vaccine.

The time between vaccination and presentation of a clinical image ranged from few days to several months. In cases of classic psoriasis flares, recorded time reports occurred in 57 studies, with the smallest value being 1 day and the largest being 90 days. The median value of post-vaccination period is 8 days in flares, 7 days in new-onset cases, and 7 days in subtype change. The Kruskal–Wallis H-test indicated that there is a non-significant difference in the post-vaccination period between the different groups (*p* = 0.993). When investigating the relation between mRNA vaccines and time of plaque psoriasis flare, the median value for mRNAs is 8 days, while for viral vectors it is 8.5 days, showing almost no difference between the two different vaccine subtypes. Also, no statistically significant difference was found between the timing of the flares between the first and second performance of Mann–Whitney testing (*p* = 0.440) while the median of the first dose-related flare timing is 10 days, while for the second dose it is 7.5 days.

It is worth mentioning that the values about timing tend to be scattered, with long time periods reported. Though cautiously, the authors of those reports attributed the flare to vaccination against COVID-19, as there was no other triggering factor reported in the medical history of the patients. In Table 5, there is a summary of our findings that in combination with Table 1 gives a completed view of the post-COVID-19 vaccination characteristics of the flare.

Concerning non-COVID-19 vaccine psoriasis contributors, there were profoundly fewer results in the categories we focused on, as we identified only nine cases. There were six plaque psoriasis flares, one new-onset, and two psoriasis subtype modifications (pustular psoriasis and guttate forms). The only new-onset case reported was due to BCG vaccination, while other types of vaccines were reported as well, such as influenza, diphtheria–tetanus (TD), and pneumococcal polysaccharide vaccine (PPSV23) (Table 6). As in the case of COVID-19 vaccinations, there were examples of patients with clinical presentation who were under biological treatment. Also, time of occurrence after vaccination ranged from 1 to 20 days for the flares, while the longest period that was observed was 30 days for the new-onset psoriasis promoted by BCG vaccination and the guttate flare in the context of plaque psoriasis triggered by vaccination against the flu (Chiroflu (anti-H1N1, H3N2 and B)) (Table 6). In the case of Grafanaki et al. [71], we detected a combined effect of viruses against influenza and pneumococcus. However, because the symptoms occurred after influenza, inactivated, split virus, or surface antigen vaccine (Vaxigrip), we considered the vaccine as the main flare etiology.

Concerning exposome traits, the findings are less relevant compared to the focus on the dynamics of the psoriasis flare. One study [50] included information about past infection with COVID-19 (infectomics aspect), finding a very high percentage of association of psoriasis exacerbations with prior infection of the respective infection. Findings for gender and age correlations were included in most cases, while the presence of comorbidities was investigated by one study, with no association found [76]. Finally, a study found that vitamin D deficiency, a vitamin well-known for its anti-inflammatory actions, predisposed patients to flare after vaccination, resulting in an observation that patients vaccinated in summer are less vulnerable to a psoriasis flare-up [77] (Table 7).

Concerning gender distribution, no difference seems to be observed, as from psoriasis flare groups, 32 female and 39 male patients were included, while in the new-onset group, 5 female/6 male patients were included, and for pustular psoriasis, 5 female/4 male patients were reported. In the non-COVID-19 vaccination group, a sex distribution difference was not reported (psoriasis flare group with 3 male and 3 female patients (Table 4)). It is worth mentioning that in the male group (5 cases with over 30 days of post-vaccination flare), long post-vaccination periods before a flare were reported (Table 2), while in the female group, only 2 patients were reported with a psoriasis flare incidence beyond 20 days (1 of which is a case of a female patient with a psoriasis flare including scalp, elbow, knee, and thigh 90 days after the second dose of Moderna vaccine [43]). The median time of female patients’ exacerbation after vaccination was 7 days, while the respective median time for male patients was 11 days (no statistical significance found, *p* = 0.08). Also, there is no significant statistical difference in the gender distribution in the patients that developed flares within a week post-vaccination (7 days) (*p* = 0.12).

The age range of psoriasis flare is from 18 to 82, while in the new-onset psoriasis, the youngest patient detected was 12 years old and the oldest was 82 years old. The age reports in each group are scattered, with most patients appearing to be elderly and few reports of young adults. The mean age for a psoriasis flare is 54.69 ± 13.49 years old, for new-onset it is 60.84 ± 21.35 years old, and for pustular modification it is 49.78 ± 20.27 years old (no statistical significance by ANOVA test, *p* = 0.4392). Comparing between the age group over 60 years old and time of psoriasis exacerbation time of occurrence, patients under 60 years of age presented median time of 7 days after vaccination, while those over 60 years old presented a median time period of 9.5 days (no statistical difference, *p* = 0.083). The age reports about the non-COVID-19 vaccines were also dispersed (Table 6).

Comorbidities are another aspect the exposome that needed to be assessed. Comorbidities ranged from chronic diseases, such as hypertension (six cases) and obesity and dyslipidemia (seven cases), skin disorders, such as atopic dermatitis and cancer incidence, such as myeloma and hepatocellular cancer (Table 2, Table 3 and Table 4). Nine patients presented a disturbed metabolic profile. Heavy smoking was reported in two cases of psoriasis flares and one of new-onset disease [40,41,45], while infectomics, including COVID-19 infection, apart from study of Wei et al. [76], was reported in a new-onset case [40] (Table 2, Table 3 and Table 4).

Reports for psoriasis flares come from all around the world, including both Caucasian and non-Caucasian populations. More precisely there were 11 Asian patients, 2 Hispanic patients, and 0 African patients (13 skin of color patients) reported, in comparison to 48 Caucasian patients. Most patients came from Greece and Italy (Table 8). Also, concerning PAF, which as discussed in the introduction is an indicator of UV exposure comparison between regions, Table 2, Table 4 and Table 5 included PAF indexes of melanoma cases as presented in [36]. A total of 37 patients were identified from countries with a PAF exceeding 0.50, and an additional 34 patients were from countries where the PAF was above 0.50. This suggests that UV intensity does not significantly impact the relationship between vaccinations and psoriasis.

## 4. Discussion

The variation in the patient population experiencing flares, coupled with the widespread use of vaccines, has the potential to make vaccine-triggered episodes an excellent example for evaluating exposome factors. This scenario allows for the examination of the delicate balance between protective and exacerbating factors. It also provides an opportunity to explore how this equilibrium, particularly when influenced by trigger factors, like vaccines, can be reversed, potentially leading to a flare. However, it remains to be seen in larger prospective studies whether vaccination is really a good and valid example for psoriasis exposome investigation. Nevertheless, our review highlights several aspects of clinical relevance, which cannot be underestimated. Analyzing how vaccination, as part of the external exposome, interacts with other exposome parameters to push towards a flare, can provide insights into the complex relationship between environmental exposures, considering factors, such as vaccine type, timing, and individual characteristics. This approach may have implications for personalized interventions and treatment strategies. Indeed COVID-19 vaccinations have been proved to trigger oxidative stress [78] and alter the microbiome [79] and metabolic profile [80]. Those external exposome alterations in combination with other characteristics of patients, such as age or comorbidities, can produce a hyperinflammation state, which is highlighted by most studies as the main mechanism through which vaccination can produce the flare [70]. Due to controlled exposure of the triggering factor and the well-defined timing of the vaccination and the onset of psoriasis flares, this approach allows for a clearer examination of cause-and-effect relationships.

However, the biological complexity implicated both in psoriasis and COVID-19 vaccination should be taken into consideration. The hyperinflammation state produced seemed to be initiated by molecular mimicry and immune cross-reaction [81], and the resulting induction of innate and adaptive immune responses that will finally promote a type of cytokine storm [7]. The evidence supporting the cytokine production theory is reinforced by the protective effect of biological therapy against flares. Additionally, the utilization of biological modalities that inhibit cytokine production and action [59], including treatments, like Anakinra (anti-IL1), can be used for managing mild to severe psoriasis exacerbations [68]. However, there are no data on cytokine modifications after COVID-19 vaccinations in psoriasis patients to comprehend cytokine regulation after vaccination in this specific population, and most studies depend on findings on other COVID-19 vaccination related adverse effects, such as myocarditis [3]. In addition to the biological complexity, there are various factors that constrain the suitability of vaccination as an example to assess the psoriasis exposome. These factors include the presence of diverse vaccines, formulations, their impact on different patient populations, and potential ethical considerations that may arise.

### 4.1. Psoriasis Flare Characteristics and COVID-19 Vaccinations

Comparing data from COVID-19 and non-COVID-19 vaccines seem to be impractical due to the limited cases of the second group (Table 2, Table 3, Table 4 and Table 6). Pustular psoriasis modification deriving from plaque psoriasis was reported more frequently compared to non-COVID-19 vaccinations, as there was only one case following pneumococcal polysaccharide vaccine (PPSV23) [71]. It is worth mentioning that our review focused on plaque psoriasis course after vaccination, so guttate new-onset psoriasis, which was the most common subtype after non-COVID-19 vaccination, is not further discussed. TD and BCG vaccines are both mRNA vaccines. Studies have demonstrated that proteins produced in response to these vaccines stimulate the production of IL6, which in turn, fosters the formation of Th1 and Th17 cells, initiating the release of subsequent cytokines that significantly contribute to the development of the epidermal changes observed in psoriasis exacerbations [82,83].

The second dose of mRNA vaccine was the most common flare contributor combination, meaning that repeated exposure to a triggering factor increases the probability of a flare. This fact can be attributed to the existence of memory T cells residing in the skin, which has been considered as the major driver of psoriasis relapse, as well as innate immune nonimmune cells with inflammatory memory produced in the first dose. Also, we observed that flares occurring after the second dose happened earlier than the respective one after the first dose [84].

Also, our review showed that there is no significant difference in time periods of post-vaccine cutaneous manifestations between plaque psoriasis flares, new-onset plaque psoriasis, and plaque psoriasis modifications to another type of psoriasis, as well as time periods of post-vaccine cutaneous manifestations between the first and second doses. This observation is mostly attributed to the small sample size in the cases of the new-onset and plaque psoriasis form change categories. Also, the three categories share common pathophysiological mechanisms affecting the skin. In the review of Nune et al. on new-onset rheumatic immune-mediated inflammatory diseases after COVID-19 vaccinations, the authors reported that the onset time of symptoms after taking the COVID-19 vaccine is short (a mean time of 10.6 days) while the most common rapid new-onset COVID-19 vaccination-related disease is myocarditis [85]. This difference in time onsets between myocarditis and psoriasis can be attributed to variation in the immune responses triggered by vaccination in terms of the organs or tissues affected and the pathways activated, meaning that the process of the skin flare can be slower compared to other organs.

### 4.2. Psoriasis Flare Characteristics, COVID-19 Vaccinations, and Exposome Factors

Psoriasis is commonly recognized as a complex disorder influenced by the interplay of genetic predisposition and environmental risk factors; therefore, genetic factors should always be considered when flares and new-onsets of the disease are in the spotlight, as psoriasis is a disease with a strong genetic component. However, there is currently only one study that integrated psoriasis vaccinalis with genetic testing results. In the case of flares, an Asian study focusing on the specific HLA-C genotype, an allele whose genotype has been linked with different psoriasis severity outcomes and subtypes, found the allele to have no correlation with worsening skin manifestations [76]. Due to the different frequency of those allele genotypes in the Caucasian populations [86], limitations in the generalizability of this conclusion are applicable. A further study in the case of new-onset psoriasis would be interesting, as individuals with advanced age, such as 82 years old (Table 3) appeared in this group, which means that no other triggering factor has been present throughout their lifetime apart from the COVID-19 vaccination that activated genes associated with psoriasis. Numerous polymorphisms in genes associated with inflammatory cytokines which render individuals more or less susceptible to the signaling cascade in the inflammatory pathway should also be investigated.

Oxidative stress is another factor that combines psoriasis and COVID-19 vaccinations. Many studies have proven that psoriasis patients are characterized by both systemic and cutaneous disturbed oxidative stress parameters. Also, disturbed oxidative stress markers, namely nitric oxide (NO) and oxidative stress index, were observed in vaccine-induced pericarditis and myopericarditis [78]. Oxidative stress as a pathway of psoriasis flares can also be supported by the fact that patients with vitamin D deficiency are more prone to exacerbation following vaccination, as vitamin D is a hormone with well-known antioxidative actions [77]. COVID-19 vaccinations have also been proved to affect the microbiome [79] and metabolics, especially purine metabolism [80]. Those inner parameters (oxidative stress, microbiome, metabolics) are already disturbed in the context of other chronic diseases and comorbidities, such as diabetes, obesity, and hypertension. The most common comorbidities found in our review that predispose patients to a flare are obesity and hypertension. Hypertension was also the top comorbidity found by the study of Huang and Tsai, but no association with exacerbation was found [76].

Gender and age were two parameters whose correlation with psoriasis flares were exclusively studied. All but one study [41] did not find differences in gender distribution. Megna et al. observed a dominance of male gender in their patients. However, this was a case series with a limited number of patients and was one of the first studies trying to elucidate the COVID-19 psoriasis vaccinalis concept. Due to the inhibitory effect of estrogens on the production of psoriasis-related cytokines, like IL-1β and IL-23, by neutrophils and dendritic cells, the conclusion that female gender is protective was formed [87]. However, consequent studies adding more cases of psoriasis flares in the male population were reported. To our knowledge, the review by Potestio et al. [14] was the last one about COVID-19 vaccination and psoriasis published and found a male–female distribution of new-onset at 10: 13 and for psoriasis flares at 61: 46. In our review, in which we focused only on flares and new-onsets that derived from plaque psoriasis, and in which we further stratified psoriasis subtypes modification, we found a male–female distribution of new-onset at 6:5 and psoriasis flares at 39:32. The main differences in new-onset disease are attributed to the fact that the study of Potestio et al. [14] included guttate, pustular, nail, and annular new-onset psoriasis cases. We also found that also there were more cases of male patients with long post-vaccination period of flare than women (period over 30 days) as well as more cases of female patients whose post-vaccination period flare occurrence did not exceed 7 days (F: 19, M: 11) meaning that female patients tend to have the flare-up more quickly after the trigger. This observation needs to be studied with more patients and with different triggering factors. Both our review report and the review by Potestio et al. [14] found the mean age of psoriasis flare patient to be 54 years old. Concerning the new onset, we found the mean age to be 60 years old, which is in accordance with the two peak incidence modes of new-onset psoriasis mode, according to which 20–30 and over 50 years of age are the main new-onset psoriasis occurrence age range [88]. Also, age and time of onset were not correlated.

Genetic variations as well as cultural and socioeconomic factors all play a role in such differences and have important implications for the psoriasis flares in skin of color patients [89]. We found 13 patients of skin of color (18%). This low percentage may be more attributable to healthcare disparities and less to the immune response differences in Caucasian and non-Caucasian skin. The only environmental factor which could be assessed was the UV radiation using the melanoma case PAF index calculated by a previous study [34]. A study suggested that vaccinations in summer months protected against flare-ups, mainly due to the immunosuppression of UV on skin. Taking into consideration the countries that case reports and cases series originated from and its PAF index, we found no differences between patients with flares coming from high (over 0.50) and low PAF index (lower than 0.50) regions. However, the extent of immunosuppression from UV depends on numerous factors related to the duration of photoexposure, such as occupation, dressing and lifestyle [90]. Infectomics, including, particularly, the medical history of COVID-19 infection, should have been more focused on by studies, as only one case series reported in their study that 83.3% of the patients with psoriasis exacerbation had a prior COVID-19 infection [43]. This finding maybe attributed to the same concept of inflammatory memory reported in the relation between the first and second doses of vaccination. Other exposome factors were taken into consideration, such as stress. Indeed, Viswanathan et al. [91], in their murine study, outlined how the stress-induced rise in leukocyte trafficking to immune activation sites, triggered by vaccination, could have beneficial effects in enhancing immunoprotection, while it may also play a simultaneous role in exacerbating autoimmune diseases, such as psoriasis.

Our study presents endogenous limitations. Firstly, there are categories presented in which the relevant studies and cases did not reach a statistically significant result, mainly due to the limited number of studies found, and, secondly, only one medical database was used (PubMed). Also, the concept of the exposome is new, with limited raw data and, therefore, more studies about the psoriasis exposome are anticipated to be published, giving new data that may not be included in the review. Finally, the term “exposome” is considered complex because it encompasses a broad and intricate concept and, as a result, the study and understanding of the interplay between various exposures and their combined impact on health is challenging.

## 5. Conclusions

There are advantages (controlled exposure, widespread use of vaccines) and disadvantages (no clear mechanism, many brands of vaccines) when considering COVID-19 vaccination as a triggering factor for plaque psoriasis flares in order to assess the psoriasis exposome. Our review consolidates reported characteristics of psoriasis flares following vaccinations and exposome traits of patients who experienced flares. Additionally, it incorporates new data, primarily derived from the combination of exposome and vaccine-triggered episode characteristics while it compares them with non-COVID-19 vaccines. We also conducted a comparison among flares, new-onset cases, and psoriasis subtype modifications concerning age and gender distribution, post-vaccination time, and treatment status. The findings reveal no significant differences, suggesting that psoriasis is impacted similarly at each stage when a triggering factor, such as vaccine-induced immune response, occurs.

## Figures and Tables

**Table 1:** Main findings of studies on plaque psoriasis flares associated with vaccinations (COVID-19 and non-COVID-19 vaccines).

(1) Individuals treated with biologics face a reduced likelihood of psoriasis flares after COVID-19 vaccination compared to other psoriatic patients [15], while patients receiving apremilast were less likely to experience psoriasis flares following COVID-19 vaccination [16].
(2) Duration of psoriasis disease may not be a predisposing factor for a psoriasis flare-up following COVID-19 vaccinations, as in a study the range of psoriasis duration in the cases was from 2 years to 54 years [17].
(3) The exacerbation of psoriasis or new-onset psoriasis occurrence has been linked to all COVID-19 vaccines, including mRNA vaccines, such as those developed by Moderna and BioNTech/Pfizer, which were often connected to subsequent episodes of psoriasis [18].
(4) Psoriasis onset was reported following the first, second, and third vaccine doses, with the second dose predominantly linked to psoriasis flares; delayed onset occurred within 2 to 21 days for new-onset cases and 1 to 90 days for flare-ups [18].
(5) All of the cases have been successfully treated, while the overall benefit–risk profile of COVID-19 vaccination does not justify vaccination avoidance.
(6) The H1N1 influenza vaccines, which were used in the 2009–2010 season, have the potential to trigger the development of psoriasis [19].
(7) 1.5% of cases from a cross-sectional retrospective study reviewing the records of 63 Brazilian patients reported psoriasis worsening after receiving the vaccine against yellow fever [20].
(8) In the case of non-COVID-19 vaccines, new-onset psoriasis occurrences are rare, while the guttate form was the most frequent psoriasis subtype that occurred after vaccination, different from COVID-19 vaccines.

**Table 2:** Characteristics of each flare as well as characteristics of the plaque psoriasis patient that experienced the exacerbation. (NM/NO: not mentioned/no, M: male, F: Female, PsA: psoriasis arthritis, PAF: population attributable fraction (PAF index) of new melanoma cases reported by 2012 data, COPD: chronic obstructive pulmonary disease).

Study	Under Biological Treatment	Days after Vaccination	Dose	Vaccine Subtype	Additional Symptoms	Sex (Hormonal Impact)	Age	Skin Type Ethnicity	Comorbidities	UV Exposure (Melanoma Cases PAF According to Region)
[42]	Brodalumab	30	2nd	mRNA	NM/NO	F	43	Japan (Asian)	Dyslipidemia, hyperuricemia, and obesity	0.74
[43]	NM/NO	20	1st	Viral vector	NM/NO	Μ	54	India (Asian)	NM/NO	0
[43]	NM/NO	3	1st	Viral vector	NM/NO	M	55	India (Asian)	NM/NO	0
[43]	NM/NO	2	1st	Viral vector	NM/NO	F	43	India (Asian)	NM/NO	0
[44]	Ixekizumab	5	2nd	mRNA	NM/NO	F	58	Germany (Caucasian)	NM/NO	0.86
[44]	Ixekizumab	7	2nd	mRNA	Pruritus	F	54	Germany (Caucasian)	NM/NO	0.86
[45]	NM/NO	7	2nd	Inactivated virus	Pruritus	F	18	Iran (Asian)	NM/NO	0.03
[46]	NM/NO	7	2nd	mRNA	NM/NO	M	53	Belgian (Caucasian)	Hypertension, diabetes mellitus, thrombo-embolism, and chronic obstructive lung disease	0.85
[47]	NM/NO	42	3nd	mRNA	NM/NO	M	64	Turkey (Asian)	NM/NO	0.4
[48]	NM/NO	10	1st	mRNA	NM/NO	F	30	Spain (Hispanic)	NM	0.761
[49]	NM/NO	4	1st	mRNA	NM/NO	M	58	Mexico (Hispanic)	Hypertension, methicillin-resistant Staphylococcus aureus osteomyelitis, untreated hepatitis C	0.32
[50]	NM/NO	62	2nd	mRNA	NM/NO	M	76	USA (NM)	NM/NO	0.905
[50]	NM/NO	21	2nd	mRNA	NM/NO	M	69	USA (NM)	NM/NO	0.905
[50]	NM/NO	6	2nd	mRNA	NM/NO	F	68	USA (NM)	NM/NO	0.905
[50]	NM/NO	60	2nd	mRNA	NM/NO	M	67	USA (NM)	NM/NO	0.905
[50]	NM/NO	7	1st	mRNA	NM/NO	F	52	USA (NM)	NM/NO	0.905
[50]	NM/NO	90	2nd	mRNA	NM/NO	F	27	USA (NM)	NM/NO	0.905
[41]	NM/NO	5	2nd	mRNA	NM/NO	M	55	Italy (Caucasian)	NM/NO	0.817
[41]	NM/NO	6	2nd	mRNA	NM/NO	M	49	Italy (Caucasian)	NM/NO	0.817
[41]	Secukinumab	10	1st	Viral vector	NM/NO	M	45	Italy (Caucasian)	NM/NO	0.817
[41]	Adalimumab	12	2nd	mRNA	NM/NO	M	61	Italy (Caucasian)	NM/NO	0.817
[41]	NM/NO	8	2nd	mRNA	NM/NO	M	62	Italy (Caucasian)	NM/NO	0.817
[41]	NM/NO	8	2nd	mRNA	NM/NO	F	70	Italy (Caucasian)	NM/NO	0.817
[41]	Guselkumab	7	2nd	Viral vector	NM/NO	F	39	Italy (Caucasian)	NM/NO	0.817
[41]	Secukinumab	5	2nd	mRNA	NM/NO	M	58	Italy (Caucasian)	NM/NO	0.817
[41]	NM/NO	10	2nd	Viral vector	NM/NO	F	55	Italy (Caucasian)	NM/NO	0.817
[41]	Etanercept	14	1st	mRNA	NM/NO	M	59	Italy (Caucasian)	NM/NO	0.817
[40]	NM/NO	8	2nd	Viral vector	Nail lesions	F	69	Greece (Caucasian)	NM/NO	0.384
[40]	NM/NO	10	2nd	mRNA	NM/NO	F	82	Greece (Caucasian)	NM/NO	0.384
[40]	NM/NO	6	2nd	mRNA	NM/NO	F	62	Greece (Caucasian)	NM/NO	0.384
[40]	NM/NO	7	2nd	mRNA	NM/NO	M	73	Greece (Caucasian)	NM/NO	0.384
[40]	Risankizumab	22	1st	Viral vector	Nail lesions	M	66	Greece (Caucasian)	NM/NO	0.384
[40]	NM/NO	6	2nd	Viral vector	NM/NO	F	64	Greece (Caucasian)	NM/NO	0.384
[40]	NM/NO	32	1st	Viral vector	NM/NO	M	69	Greece (Caucasian)	NM/NO	0.384
[40]	NM/NO	9	2nd	mRNA	NM/NO	M	81	Greece (Caucasian)	NM/NO	0.384
[40]	Ixekizumab	10	2nd	mRNA	Nail lesions	M	49	Greece (Caucasian)	NM/NO	0.384
[40]	NM/NO	7	2nd	mRNA	Nail lesions	F	55	Greece (Caucasian)	NM/NO	0.384
[40]	Guselkumab	7	2nd	Viral vector	NM/NO	F	64	Greece (Caucasian)	NM/NO	0.384
[51]	Ustekinumab	7	1st	Viral vector	NM/NO	F	31	Taiwan (Asian)	NM/NO	0.504
[52]	NM/NO	60	1st	mRNA	Pruritus and PsA	M	51	Turkish (Asian)	Diabetes	0.403
[52]	NM/NO	30	2nd	Inactivated virus	NM/NO	M	52	Turkish (Asian)	NM/NO	0.403
[53]	Nivolumab	7	1st	mRNA	Pruritus	M	65	USA (NM)	Hepatocellular carcinoma	0.905
[54]	Risankizumab	1	2nd	mRNA	Pruritus and PsA	M	34	Japan (Asian)	NM/NO	0.704
[55]	NM/NO	7	1st	Viral vector	NM/NO	F	53	India (Asian)	NM/NO	0
[56]	NM/NO	1	2nd	mRNA	NM/NO	M	46	Caucasian	NM/NO	0.669
[57]	Secukinumab	10	2nd	mRNA	NM/NO	F	34	Greece (Caucasian)	Celiac disease and Hashimoto	0.384
[57]	NM/NO	14	1st	Viral vector	NM/NO	M	61	Greece (Caucasian)	hypertension	0.384
[57]	NM/NO	10	2nd	mRNA	NM/NO	F	45	Greece (Caucasian)	Psoriasis arthritis	0.384
[57]	Adalimumab	2	2nd	mRNA	NM/NO	F	56	Greece (Caucasian)	Crohn, COPD, dyslipidemia	0.384
[57]	Adalimumab	20	1st	mRNA	NM/NO	F	53	Greece (Caucasian)	Crohn, arthralgia	0.384
[57]	Adalimumab	3	1st	mRNA	PsA	F	56	Greece (Caucasian)	Arthralgia	0.384
[57]	Secukinumab	7	1st	mRNA	NM/NO	F	34	Greece (Caucasian)	NM/NO	0.384
[57]	NM/NO	4	2nd	mRNA	NM/NO	F	61	Greece (Caucasian)	Rheumatoid arthritis, hypothyroidism	0.384
[57]	Ustekinumab	20	1st	mRNA	NM/NO	F	66	Greece (Caucasian)	PsA, type 2 diabetes, essential hypertension, dyslipidemia	0.384
[57]	NM/NO	20	2nd	mRNA	NM/NO	F	67	Greece (Caucasian)	Psoriatic arthritis	0.384
[57]	NM/NO	20	2nd	mRNA	NM/NO	M	56	Greece (Caucasian)	Acute coronary disease	0.384
[57]	NM/NO	25	2nd	mRNA	NM/NO	M	51	Greece (Caucasian)	Psoriatic arthritis	0.384
[17]	NM/NO	NM	3rd	mRNA	NM/NO	F	40	Italy (Caucasian)	NM/NO	0.817
[17]	NM/NO	NM	2nd	mRNA	NM/NO	M	50	Italy (Caucasian)	NM/NO	0.817
[17]	NM/NO	NM	2nd	mRNA	NM/NO	M	25	Italy (Caucasian)	NM/NO	0.817
[17]	NM/NO	NM	3rd	mRNA	NM/NO	F	40	Italy (Caucasian)	NM/NO	0.817
[17]	NM/NO	NM	2nd	mRNA	NM/NO	M	53	Italy (Caucasian)	NM/NO	0.817
[17]	NM/NO	NM	2nd	mRNA	NM/NO	M	50	Italy (Caucasian)	NM/NO	0.817
[17]	NM/NO	NM	2nd	mRNA	NM/NO	M	38	Italy (Caucasian)	NM/NO	0.817
[17]	NM/NO	NM	1st	mRNA	NM/NO	M	56	Italy (Caucasian)	NM/NO	0.817
[17]	NM/NO	NM	2nd	mRNA	NM/NO	M	50	Italy (Caucasian)	NM/NO	0.817
[17]	NM/NO	NM	3rd	mRNA	NM/NO	M	78	Italy (Caucasian)	NM/NO	0.817
[17]	NM/NO	NM	3rd	mRNA	NM/NO	F	67	Italy (Caucasian)	NM/NO	0.817
[17]	NM/NO	NM	2nd	mRNA	NM/NO	M	53	Italy (Caucasian)	NM/NO	0.817
[17]	NM/NO	NM	2nd	mRNA	NM/NO	M	61	Italy (Caucasian)	NM/NO	0.817
[17]	NM/NO	NM	3rd	mRNA	NM/NO	M	76	Italy (Caucasian)	NM/NO	0.817

**Table 3:** Characteristics of new-onset psoriasis as well as characteristics of the plaque psoriasis patient that experienced the symptoms. (NM/NO: not mentioned/no, M: male, F: Female, PAF: population attributable fraction (PAF index) of new melanoma cases reported by 2012 data, COPD: chronic obstructive pulmonary disease).

Study	Under Biological Treatment	Days after Vaccination	Dose	Vaccine Subtype	Additional Symptoms	Sex	Age	Skin Type Ethnicity	Comorbidities	UV Exposure (Melanoma Cases PAF According to Region)
[44]	NM/NO	7	2nd	mRNA	PsA	F	50	Germany (Caucasian)	NM/NO	0.86
[45]	NM/NO	NM	1st	Inactivated virus	Pruritus	M	34	Iran (Asian)	Chronic urticaria	0.03
[45]	NM/NO	4	1st	Inactivated virus	Pruritus	M	50	Iran (Asian)	Arthritis	0.03
[58]	NM/NO	7	1st	Viral vector	NM/NO	M	51	Vietnam (Asian)	Atopic dermatitis and hypertension	0
[58]	NM/NO	30	3rd	mRNA	NM/NO	F	68	Vietnam (Asian)	Hypertension	0
[58]	NM/NO	30	1st	mRNA	Pruritus	M	73	Vietnam (Asian)	COPD, hypertension, diabetes	0
[48]	NM/NO	6	2nd	mRNA	NM/NO	M	72	Spain (Hispanic)	Myeloma	0.761
[50]	NM/NO	24	2nd	mRNA	NM/NO	M	89	USA(NM)	NM/NO	0.905
[43]	NM/NO	10	2nd	Viral vector	NM/NO	M	65	India (Asian)	NM/NO	0
[59]	NM/NO	21	1st	mRNA	NM/NO	F	12	Iran (Asian)	NM/NO	0
[60]	NM/NO	1	2nd	mRNA	NM/NO	F	63	China (Asian)	NM/NO	0.504
[61]	NM/NO	7	1st	mRNA	NM/NO	F	82	Italy (Caucasian)	Hypertension, anemia	0.817

**Table 4:** Characteristics of psoriasis subtype modification as well as characteristics of the plaque psoriasis patient that experienced the symptoms. (NM/NO: not mentioned/no, M: male, F: Female, PsA: psoriasis arthritis, PAF: population attributable fraction (PAF index) of new melanoma cases reported by 2012 data).

Study	Psoriasis Subtype Modification	Under Biological Treatment	Days after Vaccination	Dose	Vaccine Subtype	Additional Symptoms	Sex	Age	Skin Type Ethnicity	Comorbidities	UV Exposure (Melanoma Cases PAF According to Region)
[44]	Guttate	NM/NO	7	2nd	mRNA	PsA	M	35	Germany (Caucasian)	NM/NO	0.86
[44]	Guttate	Ustekinumab	10	2nd	mRNA	PsA	M	62	Germany (Caucasian)	NM/NO	0.86
[58]	GEP	NM/NO	60	2nd	mRNA	NM/NO	F	30	Vietnam (Asian)	NM/NO	0
[58]	GEP	NM/NO	7	2nd	mRNA	NM/NO	F	45	Vietnam (Asian)	NM/NO	0
[62]	Guttate	NM/NO	7	3rd	mRNA	NM/NO	F	80	Singapore (Asian)	NM	0
[41]	Guttate	Ixekizumab	9	2nd	mRNA	NM/NO	M	47	Italy (Caucasian)	NM/NO	0.817
[40]	Guttate	NM/NO	3	2nd	Viral vector	NM/NO	F	63	Greece (Caucasian)	NM/NO	0.384
[63]	Pustular	NM/NO	5	1st	mRNA	NM/NO	M	40	USA (Caucasian)	obesity, hypertension, depression, anxiety	0.905
[64]	Pustular	NM/NO	4	1st	Inactivated virus	NM/NO	M	73	Turkey (Asian)	Hypertension	0.403
[65]	Pustular	NM/NO	4	1st	Inactivated virus	NM/NO	M	21	India (Asian)	NM/NO	0
[66]	Pustular	Ustekinumab	10	2nd	mRNA	NM/NO	F	47	Italian (Caucasian)	Obesity	0.817
[67]	Pustular	NM/NO	7	1st	mRNA	NM/NO	F	18	Japan (Asian)	NM/NO	0.704
[68]	Pustular	NM/NO	5	2nd	mRNA	NM/NO	F	56	Greece (Caucasian)	Obesity	0.384
[17]	Pustular	Ustekinumab	NM	2nd	mRNA	NM/NO	F	73	Italy (Caucasian)	NM/NO	0.817
[69]	GEP	NM/NO	28	2nd	mRNA	Pruritus	M	53	USA (Caucasian)	NM/NO	0.905
[70]	Pustular	NM/NO	30	NM	mRNA	NM/NO	M	57	Italy (Caucasian)	NM/NO	0.817
[70]	Pustular	NM/NO	30	NM/	mRNA	NM/NO	F	63	Italy (Caucasian)	NM/NO	0.817

**Table 5:** Summary of the post-vaccination psoriasis characteristics added by our review.

(1) Pustular psoriasis manifestations are more common after COVID-19 vaccines compared to other types of vaccines.
(2) Biological treatment does not provide complete protection but reduces the likelihood of an exacerbation.
(3) The most common vaccine subtype contributor for new-onset psoriasis and a pustular psoriasis change profile is the mRNA vaccine (66.7%) while the second dose of an mRNA vaccine was the most common flare contributor combination.
(4) There is no significant difference in time periods of post-vaccine cutaneous manifestations between plaque psoriasis flares, new-onset plaque psoriasis, and plaque psoriasis modifications to another type of psoriasis.
(5) There is no significant difference in time periods of post-vaccine cutaneous manifestations between the first and second dose. However, it seems that a flare occurring after the second dose happens earlier than the respective one after the first dose.

**Table 6:** Characteristics of each flare provoked by non-COVID-19 vaccines as well as characteristics of the plaque psoriasis patient that experienced the exacerbation. (NM: not mentioned, M: male, F: Female).

Study	Psoriasis Characteristics	Under Biological Treatment	Occurrence after Vaccination	Type of Vaccination	Sex	Age
[72]	Psoriasis subtype modification (pustular psoriasis)	NM/NO	5	PPSV23	F	70
[71]	Psoriasis flare	NM/NO	20	Vaxigrip	M	55
[10]	Psoriasis flare	Adalimumab	1	Chiroflu (anti-H1N1, H3N2 and B)	M	41
[10]	Psoriasis flare	NM/NO	7	Chiroflu (anti-H1N1, H3N2 and B)	F	70
[10]	Psoriasis flare	Secukinumab	1	Chiroflu (anti-H1N1, H3N2 and B)	F	55
[10]	Psoriasis subtype modification (guttate)	Guselkumab	30	Chiroflu (anti-H1N1, H3N2 and B)	F	67
[73]	Psoriasis flare	NM/NO	NM/NO	Fluarix influenza vaccine	F	30
[74]	Psoriasis flare	NM/NO	7	Td vaccine	M	50
[75]	New-onset psoriasis	NM/NO	30	BCG vaccine	F	6

**Table 7:** Main findings of the exposome characteristics of patients with plaque psoriasis flares associated with COVID-19 vaccinations.

Genomics: No specific Human leukocyte antigen-C(HLA-C) genotype is found to be related to worsening of skin manifestations after COVID-19 vaccination [76].
Infectomics: In their study 83.3% of the patients with psoriasis exacerbation had prior COVID-19 infection [50].
Male gender can be a predictive factor for a psoriasis flare-up [41].
Comorbidities do not contribute to psoriasis vaccinalis [76].
Vaccinations in summer months protected against flare-ups [77].

**Table 8:** Summary of the exposome traits presented by patients with post-vaccination psoriasis flares according to our review.

(1) In the medical literature, there are more cases of male patients with a long post-vaccination period of flares than women (period over 30 days), as well as more cases of female patients whose post-vaccination flare occurrence period did not exceed 7 days (F: 19, M: 11)
(2) There are no differences in gender distribution between plaque psoriasis flares, new-onset plaque psoriasis, and plaque psoriasis modifications to another type of psoriasis
(3) There is no correlation between age and post-vaccination time of psoriasis flare.
(4) The most reported comorbidity reported in the patients with psoriasis flares is obesity and dyslipidemia
(5) Most case series and case reports come from Greece and Italy, while only almost 18% (13 out of 71) were from non-Caucasians individuals.

## Data Availability

The data described in this study are available upon request from the corresponding author.
